# A scoping review investigating the use of exposure for the treatment and targeted prevention of anxiety and related disorders in young people

**DOI:** 10.1002/jcv2.12080

**Published:** 2022-05-20

**Authors:** Alessandra K. Teunisse, Lorna Pembroke, Maddison O’Gradey‐Lee, Megan Sy, Ronald M. Rapee, Viviana M. Wuthrich, Cathy Creswell, Jennifer L. Hudson

**Affiliations:** ^1^ Centre for Emotional Health School of Psychological Sciences Macquarie University, Macquarie Park New South Wales Australia; ^2^ Black Dog Institute University of New South Wales Randwick New South Wales Australia; ^3^ Department of Experimental Psychology and Department of Psychiatry University of Oxford Oxford UK

**Keywords:** anxiety, behaviour, internalising disorder, intervention, obsessive–compulsive disorder, therapy

## Abstract

**Background:**

Cognitive Behavioural Therapy (CBT) is the gold standard intervention for anxiety and related mental health disorders among young people; however, the efficacy of individual elements of CBT (e.g., exposure to feared stimuli) have received little scrutiny.

**Aims:**

This scoping review, informed by three stakeholder groups and a scientific advisory group, aimed to identify the nature and extent of the available research literature on the efficacy of exposure to feared stimuli, moderators of effectiveness in young people aged 14–24 years.

**Method:**

Three international stakeholder groups composed of clinicians (*N* = 8), parents/carers (*N* = 5) and youth with lived experience of anxiety (*N* = 7) provided input into study design and results. Using the PRISMA extension for scoping reviews, a search of MEDLINE/Ovid, PsycINFO, PubMed, CINAHL, SCOPUS, EMBASE, ERIC, and Health Collection (informit) was conducted using terms related to anxiety, ages 14–24, and exposure.

**Results:**

From 3508 unique abstracts, 64 papers were included for the review. While there was evidence for the efficacy of exposure as a treatment for youth anxiety disorders, fundamental gaps in knowledge of exposure in this age group were identified. Most studies examined post‐traumatic stress disorder, obsessive–compulsive disorder, and specific phobias with no randomised clinical trials uniquely evaluating exposure for the treatment of DSM‐5 anxiety disorders. Exposure was typically delivered accompanied by other anxiety management techniques. A multitude of optimisation strategies have been tested, yet only one of these effects (timing relative to sleep) showed preliminary evidence of replication.

**Conclusions:**

A systematic and theoretically driven program of research investigating the efficacy of exposure in young people and factors that moderate its efficacy, along with methods to overcome barriers for delivery, is urgently needed.

**Key points**

- Exposure is an active ingredient in treatment of anxiety disorders in young people yet significant barriers prevent wide spread delivery of this technique
- Fundamental gaps in knowledge of exposure in 14–24 years were identified in this scoping review
- Most studies examined post‐traumatic stress disorder, obsessive–compulsive disorder, and specific phobias with no randomised clinical trials uniquely evaluating exposure for the treatment of DSM‐5 anxiety disorders
- Optimisation strategies have been identified but rarely replicated in this age group
- A systematic and theoretically driven program of research investigating the efficacy of exposure in young people and factors that moderate its efficacy, along with methods to overcome barriers for delivery, is urgently needed

As anxiety and related disorders (like post‐traumatic stress disorder (PTSD) and obsessive–compulsive disorder (OCD)) are together the most prevalent and chronic group of mental disorders in adolescents and young adults (Merikangas et al., 2010), it is vital that the most efficacious aspects of treatments are identified and we develop a greater understanding of the mechanisms for change (Bittner et al., 2007). A focus on this developmental stage is of particular importance because of the high rates of anxiety disorders during this period (NHS, 2019). Cognitive Behavioural Therapy (CBT) is recommended as the first line of treatment for anxiety and related disorders in young people in numerous treatment guidelines with approximately half to two‐thirds of young people responding favourably to CBT (Higa‐McMillan et al., 2016; James et al., 2020). CBT with young people typically involves anxiety management strategies (e.g., cognitive restructuring, relaxation) followed by exposure (Whiteside et al., 2015), however, the efficacy of individual elements of CBT for youth has received little scrutiny (Whiteside et al., 2015) limiting efforts to improve both the efficacy and efficiency of treatments.

Exposure can be defined as prolonged and repeated confrontation with the feared object or situation in a systematic and controlled manner while preventing avoidance (Mobach et al., 2020) and is generally considered to be a critical component of CBT (Abramowitz et al., 2019). Exposure as a therapeutic technique was originally developed from the principles of associative learning, more specifically extinction learning (Marks, 1973). Exposure paradigms within CBT packages for anxiety disorders in young people have traditionally been based on a fear habituation model proposing that exposure allows the person to habituate to the emotion of fear (Foa & Kozak, 1986). More recently, the inhibitory learning model has proposed that exposure is successful because it establishes new memories about the feared object that compete with old memories (Craske et al., 2014). Based on this model, several strategies have been proposed to enhance the efficacy of exposure, such as violating expectancies about harm, occasional reinforced extinction, reducing safety behaviours, stimulus variability, enhancing memory consolidation, and affect labelling (Craske et al., 2014). Most empirical evaluation of these predictions has been limited to adult populations and our knowledge of the mechanisms of exposure in youth is less well‐developed. This is a serious omission given that adolescents and young adults differ from older and mature adults in several important ways including cognitive maturity (Arain et al., 2013). Some evidence has indicated that both adolescent humans and rodents exhibit poorer extinction than younger and older groups (K. D. Baker et al., 2014). Therefore, it is vital to understand if exposure is an effective approach to reduce anxiety in an adolescent age‐group.

Despite growing evidence informing the optimisation of exposure use with adults, there is a paucity of research examining strategies to improve the effects of exposure among young people. For example, treatment studies have applied exposure in many different forms such as in vivo (i.e., in real situations, not imaginal) or imaginal, graduated or flooding, one session treatment or across multiple treatment sessions, in session or out of session, and with or without coping strategies, with no comparisons of efficacy between various approaches. In most CBT packages for anxiety disorders in young people, exposure is graded and delivered both in session and out of session, and usually preceded by the introduction of coping strategies (Kendall & Peterman, 2015) despite recent evidence that these might not enhance outcomes, and may in fact impede positive treatment outcomes (Whiteside et al., 2020).

The objective of this scoping review was to identify the main sources of evidence regarding the use of exposure in the management of anxiety‐related disorders among young people aged 14–24 years. This age range was selected by the agency funding this work, the Wellcome Trust, as part of a programme to identify the active ingredients in treatment of anxiety and/or depression in young people, given the unique needs of this age group and the high prevalence of anxiety and depression during these periods of development. This scoping review focused on the evidence for the efficacy of exposure and factors that increase or decrease its effectiveness. The scoping review included studies that specifically used exposure to treat or prevent anxiety disorders (clinical and subclinical) as included in the DSM‐IV and DSM‐5. This means that even though PTSD and OCD are no longer classified as anxiety disorders in DSM‐5, they have been included, given exposure is a core component of treatment for these disorders and is commonly the primary ingredient.

There is also evidence to indicate that there are significant barriers to the delivery of CBT strategies, in particular exposure, due to a range of factors (such as clinician beliefs and confidence) resulting in young people missing out on evidenced‐based care. As discussed by Beames et al. (2021), including lived experience perspectives in reviews of mental health research may lead to an increased up‐take of treatments and increased engagement. Thus, stakeholder groups (young people, parents/carers, clinicians) were included at two points in the review process and were critical to shaping the research design and interpretation of the results.

## METHODS

### Protocol and registration

This scoping review used the six review stages as recommended by Arksey and O’Malley (2005) and followed the advice of Levac et al. (2010) as well as the PRISMA‐ScR guidelines to improve methodological and reporting quality (Tricco et al., 2018). The study protocol was registered on the Open Science Framework (DOI 10.17605/OSF.IO/4V63H).

### Search strategy

Seven databases were employed for the literature search: *PsycINFO, PubMed, CINAHL, SCOPUS, EMBASE, ERIC, and Health Collection (informit).* The search was conducted on July 13^th^, 2020 and no time limit was set, thus, the end date was 13/07/2020. Key search terms are presented in Table 1.

**TABLE 1:** Search terms

Anxiety	Population (age 14–24)	Treatment (incl. exposure)
Anxi* Agoraphobia Anxiety disorder Avoid* Generalised/generalized anxiety/GAD OCD/obsessive–compulsive disorder Panic PTSD/post‐traumatic stress disorder Selective mutism Separation anxiety Social anxiety Social phobia Specific fears Specific phobia Fear Worry Culture‐bound anxiety‐related disorders: Khyâlcap Ataque de nervios Taijin‐kyofu‐sho pelo y tata Shenjing shuairuo Koro Ghost sickness Susto Briquet's disorder Latah Occupational neurosis Psychasthenia Psychasthenic neurosis psychogenic syncope Distress* Internali* Freezing	Adolesc* College Junior Juvenile Paediatric/Pediatric Student Teen* Young AND (adult, person, people) Young* Youth School‐age Developmental differences Child Emerging adults University student Undergraduate student Minor	Behavio* experiment Behavio* therapy CBT Confront* Desensit* ERP EX/RP Expos* AND (therapy OR treatment OR intervention OR behavio*) Extinct* Flooding Graded expos* Habituation Imaginal expos* Implosive therapy In vivo expos* Interoceptive expos* Response prevention Systematic desensit* Virtual reality exposure therapy One‐session treatment Inhibitory

*Notes:* When entering these terms into the database on July 13^th^, 2020, the terms within the columns were combined with ‘OR’, and the terms between the columns were combined using ‘AND’. For example, (anx* OR worr* OR panic* etc) AND (adolesc* OR student etc.) AND (cbt OR exposure therapy etc.) Where possible, filters were applied to target empirical studies, papers in English, and population ages between 14 and 25 years.

### Eligibility criteria

Studies were eligible for inclusion if the following conditions were met: (i) mean age of participants between 13.5 and 24.4 years (regardless of age range), or if the mean age fell outside this bracket then the study provided an age‐specific analysis on a subset of individuals between 14 and 24 years, (ii) participants were selected for the study due to the presence of a current anxiety disorder (as defined by DSM‐IV or 5) or elevated anxiety symptoms, and (iii) peer‐reviewed empirical studies. Peer reviewed studies were included if they addressed questions on efficacy and/or moderators of exposure. We included studies in which the interventions or experimental conditions involved exposure as the primary ingredient (e.g., started in session 1 and comprised almost all sessions). These intervention studies needed to include a measure of anxiety‐ or fear‐related outcomes that could potentially impact upon daily function. Studies examining interventions that included exposure as part of a broader treatment package (e.g., treatment involving cognitive restructuring, relaxation, and exposure) were also included if a measure of anxiety/impact was taken before and after the introduction of exposure so that the specific effect of exposure could be extracted. However, as exposure is rarely included entirely on its own and because we were interested in the potential augmenting effect of other concurrent strategies, studies were not excluded if young people were given additional strategies to use during exposure (e.g., relaxation, cognitive restructuring). In these cases, exposure still needed to comprise most sessions and be the primary ingredient. Studies were excluded if they were narrative reviews, meta‐analyses, systematic reviews, or grey literature.

### Study selection

After papers were extracted from a search of the electronic databases, each abstract was screened by two research team members using Covidence (Veritas Health Innovation, 2020). The abstracts of these studies were retrieved, and full texts screened for inclusion by two research team members. All conflicts regarding study eligibility were discussed and resolved between AT, LP, and MO.

### Data synthesis

A coding framework was developed to extract data regarding details about the study, including date, location, sample characteristics, aim, design, details of exposure treatment, measures, and outcomes. An analysis of study quality is not typically included in a scoping review (Tricco et al., 2018).

### Stakeholder engagement

The research team met with three stakeholder groups twice. The groups comprised clinicians delivering psychological treatments (*N* = 8), parents/carers of a young person with lived experience of anxiety (*N* = 5), and youth with lived experiences of anxiety disorders (*N* = 7). The focus groups were conducted in English (via Zoom) and included participants from Australia, UK, Japan, USA, Netherlands, Germany, India, Brazil, Thailand, and Malaysia. The research project was presented to each group in the first meeting and they provided feedback based on their experiences and expertise with exposure and/or psychological therapy for anxiety disorders. The search terms were then reviewed to ensure the experiences of the stakeholders were reflected. The research team met with each group again to present the review findings and to discuss how the results reflected their experiences. A scientific advisory group (*N* = 8; for membership see acknowledgements) was engaged on two occasions to provide feedback on the search strategy and the results.

## RESULTS

**FIGURE 1:**
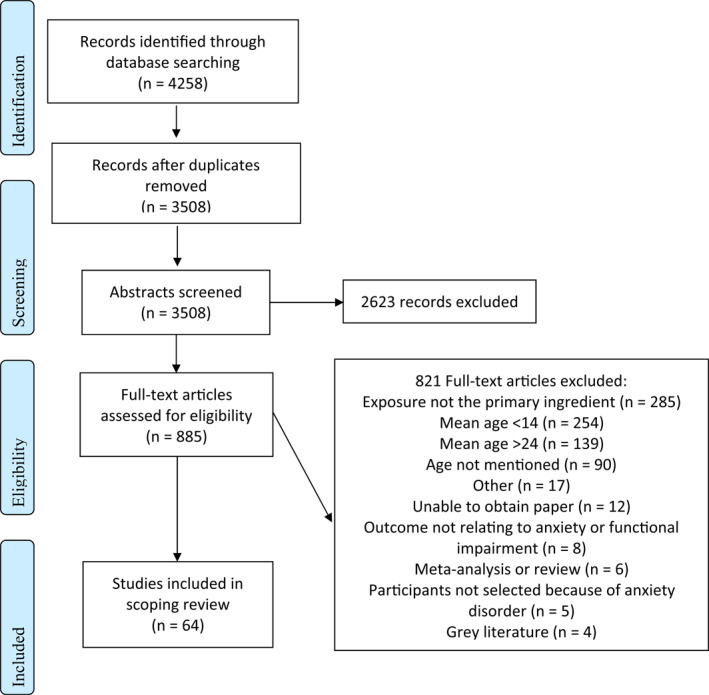
PRISMA flowchart of study selection process

Figure 1 presents the flow diagram of articles selected for the scoping review. After screening 3508 abstracts and 885 full‐text articles, 64 articles matched our criteria. The papers were diverse in methodologies, country of origin, and primary anxiety type (See Table 2). Table 3 outlines the details of the included studies such as author details, purpose of the study, and information on the sample and measures used. Table 4 specifies the types of exposure and additional elements used in each study (See Appendix S1). Across all the studies reviewed, 63% (*n* = 40) of the papers examined exposure using an in vivo format (91% of randomised controlled clinical trials (RCCTs; i.e., randomised controlled clinical trials where therapeutic doses of exposure were administered; *n* = 10) and 58% (*n* = 37) examined exposure using imaginal techniques (82% of randomised controlled clinical trials; *n* = 9). Most studies applied exposure using a gradual approach, that is, starting with less fearful situations (*n* = 39, 61%); with a high proportion of RCCTs using gradual exposure (*n* = 9; 82%). Studies less consistently examined exposure and included other skills during the program: psychoeducation (*n* = 20; 31%), relaxation (*n* = 16; 25%), or cognitive strategies (*n* = 3; 5%). Examining RCCTs specifically, 64% tested exposure with relaxation, 55% with psychoeducation about anxiety, and 9% with cognitive restructuring techniques. Only 16% (*n* = 10) of all studies reviewed used technology to assist exposures (0% of RCCTs). Only 9% of studies (*n* = 6) tested intensive exposure, that is exposure massed in a single session (at least 2 h) or a series of sessions (i.e., having more than one session a week).

**TABLE 2:** General characteristics of included studies

	Number (*N* = 64)	Percentage (%)
Date of publication		
<1990	4	6.3
1990–1999	5	7.8
2000–2009	16	25.0
2010–2019	39	60.9
Geographical locations		
Africa	3	4.7
Asia	2	3.1
Australia	2	3.1
Canada	1	1.6
Europe	15	23.4
Israel	1	1.6
USA	40	62.5
Study design		
Case reports/case series	18	28.1
Cohort studies	8	12.5
Experiments & quasi‐experiments	27	42.2
Randomised controlled clinical trials	11	17.2
Participant type		
Community and/or university students	29	45
University and school age	1	1.6
High school students	2	3.1
Patient	31	48.4
Other	1	1.6
Primary anxiety type		
OCD	15	23.4
Performance anxiety	9	14.1
PTSD	13	20.3
Social anxiety/phobia	4	6.3
Simple/specific fears/phobia	20	29.7
NA	3	6.3
Therapy context		
Individual	58	90.6
Group	6	9.4

**TABLE 3:** List of included papers

Author(s), year, and location	Purpose	No. of participants, type, and mean age	Primary anxiety type (comorbidities)	Anxiety‐/fear‐related outcome measures	No. of exposure sessions (length)^a^
Case reports^b^					
Abramowitz (2002), USA	To treat obsessive thoughts and cognitive rituals using ERP	1 patient (19 years)	OCD (MDD)	Y‐BOCS	13 sessions (unspecified)
Buchanan and Houlihan (2008), USA	To describe the multicomponent assessment and treatment of a severe phobia of earthworms	1 university student (20 years)	Simple/specific phobia—earthworms	SUDS	12 sessions (45 min per session)
				STAI	
				BAT	
Chok et al. (2010), USA	To treat a dog phobia by targeting behavioural avoidance and physiological reactivity in a child with autism and an intellectual disability	1 patient (15 years)	Simple/specific phobia—dogs (ASD, intellectual disability)	HR	18 sessions (unspecified)
Frye and Spates (2012), USA	To determine whether prolonged exposure augmented with mindfulness and emotion regulation may reduce anxiety sensitivity and aid clients to remain in treatment	1 patient (19 years)	PTSD	SUDS	8 sessions (twice weekly session of 4 × 90 min and 4 × 120 min)
				ASI	
Kitchiner (2000), Australia	To describe the treatment of a patient with PTSD using EMDR in one session	1 patient (22 years)	PTSD	SUDS	1 session (60 min)
				Fear questionnaire	
Rothbaum et al. (1995b), USA	To test the efficacy of computer‐generated virtual reality (VR) for the treatment of acrophobia (fear of heights)	1 patient (19 years)	Simple/specific phobia—acrophobia	AQ	5 sessions (35–45 min per session)
				BAT	
Newman and Adams (2004), USA	To use systematic desensitization and modelling to treat a dog phobia in a young man with learning disability	1 patient (17 years)	Simple/specific phobia—dogs (moderate learning disability)	Therapist observation and client self‐report	28 sessions (30–40 min per session)
Saigh (1987), USA	To describe the treatment of PTSD using flooding	1 patient (14 years)	PTSD	STAI	6 sessions (60 min per session)
				BAT	
Whiteside and Abramowitz (2006), USA	To describe the use of intensive ERP for the treatment of paediatric obsessive–compulsive disorder (OCD)	1 patient (14 years)	OCD	CY‐BOCS	14 daily sessions, 4 or 5 days per week over 3 weeks (unspecified)
				SCAS	
				SUDS	
Woods et al. (2000), USA	To apply ERP for treatment of compulsions in an adolescent with comorbid Tourette's Syndrome and OCD	1 patient (16 years)	OCD (Tourette's Syndrome)	SUDS	11 sessions (unspecified)
				Overt anxious behaviour (signing or rocking of head)	
Wu and Storch (2016), USA	To describe the treatment of harm‐related obsessions in a child with OCD, using a full course of family‐based ERP	1 patient (15 years)	OCD (mild secondary depressive symptoms)	CY‐BOCS	13 sessions (60 min per session)
				SUDS	
Case series					
Burton et al. (2017), UK	To describe a series of cases using a flexible protocol for the treatment of dog phobia in young people with ASD spectrum disorder and intellectual disability and severe communication impairments	5 patients (14–19 years)	Simple/specific phobia—dogs (ASD, ID)	Not available	6–25 sessions (no more than 30 min per session)
Culver et al. (2012), USA	To investigate whether the presence of additional excitatory and arousing stimuli throughout exposure facilitates long‐term fear reduction	59 university students (*M* = 19.5, 18–25)	Performance anxiety	SSPS	1 session (4 × 5 min)
				PRCS	
				BAT	
				SUDS	
Farrell et al. (2016), Australia	To examine the efficacy of an intensive ERP‐based treatment for OCD youth	10 patients (*M* = 13.6 years, SD = 1.84, 11–16)	OCD (anxiety, ADHD, ODD, specific phobia, GAD, SAD)	ADIS‐P	2 sessions (2–3 h per session)
				CY‐BOCS	
				NIHM‐GOCS & CGI	
				MASC	
Hendriks et al. (2017), Netherlands	To investigate the efficacy and safety of an intensive PE therapy for adolescents in an experimental design that controlled for time effects	10 patients (*M* = 15.90, SD = 1.52, 13–18)	PTSD (depressive disorder, psychotic features, other anxiety disorders, eating disorder, conversion disorder, ASD & alcohol dependence)	UCLA PTSD‐RI	18 sessions (90 min per session)
				SCARED	
				K‐SADS‐PL	
Iniesta‐Sepulveda et al. (2018), USA	To present a case series of nine adolescents with OCD who participated in multimodal, CBT‐based intensive outpatient or partial hospitalization programs for OCD	9 patients (*M* = 14, SD = 2.00, 11–17)	OCD (ASD)	CY‐BOCS	24‐80 sessions (*M* = 46.5)
				CIS‐C/P	
				CGI‐S	Either an intensive outpatient program (3 h/day of ERP, 5 days a week) or partial hospitalization program (4–5 h of ERP per day, 5 days a week)
				SCARED	
Marr (2012), UK	To describe the treatment of EMDR for OCD	2 patients (*M* = 21.5, 19 & 24)	OCD	SUDS	14–16 sessions (60 min per session)
				CY‐BOCS	
Whiteside et al. (2008), USA	To describe the use of an intensive 5‐day treatment for obsessive–compulsive disorder (OCD) with three adolescents	2 patients (*M* = 16, 14 & 18)	OCD	ADIS‐C	8 sessions (60 min per session over 5 days)
				CY‐BOCS	
				SCAS	
Cohort studies					
A. Baker et al. (2010), USA	To report on the determine if within session habituation (WSH) and between session habituation (BSH) require initial fear activation (IFA)	44 university students (*M* = 18.88, SD = 0.99)	Simple/specific phobia—acrophobia	BAT	2 session (average duration = 21.27 min)
				AQ—anxiety subscale	
				HR	
Fischer et al. (1998), USA	To investigate the feasibility and efficacy of group behavioural therapy in the treatment of adolescents with OCD	15 patients (*M* = 14.5, 12–17)	OCD	CY‐BOCS	7 weekly sessions (90 min per session)
Kahlon et al. (2019), Norway	To investigate the feasibility of adapting the 3‐h single session virtual reality exposure therapy (VRET) protocol for public speaking anxiety with adolescents in a non‐randomized feasibility pilot study	27 high school students (*M* = 14.22, SD = 0.64, 13–16)	Performance anxiety	PSAS	1 session (60 min per session)
				SIAS	
				SUD	
Krebs et al. (2015), UK	To examine whether i) treatment non‐response in routine clinical practice is due to failures in the delivery of treatment, and ii) patients who are treatment‐resistant will respond to treatment if adequately delivered	15 patients (*M* = 15 years and 8 months, 10–18)	OCD (depression, autism, Tourette Syndrome, social anxiety disorder, selective mutism & conduct disorder)	CY‐BOCS	8‐12 session (60 min per session)
				Experience of previous CBT Interview Schedule	
Mathes et al. (2019), USA	To examine the validity of the mental contamination task (i.e., participants are instructed to imagine a bowl of vomit for 1 min, and are then asked to describe the vomit in detail) in a sample of individuals with primary contamination symptoms and to investigate the relationship between responses to contact contamination and mental contamination tasks	88 university students (*M* = 19.03, SD = 1.79)	OCD	VOCI	3 sessions (102 min per session)
				VOCI‐MC	
				DOCS	
Morina et al. (2015), Netherlands	To investigate whether two‐session exposure to a virtual reality‐based program will lead to a reduction of social anxiety	38 university students (*M* = 22.3, SD = 5.7, 18–51)	Social anxiety/phobia	SIAS	2 sessions (30 min per session)
Riise et al. (2018), Norway	To perform a systematic replication to test the Bergen four day treatment (B4DT) in which disorder, treatment and setting remains the same, but therapists vary	41 patients (*M* = 15, SD = 1.8, 11–18)	OCD (depression, other anxiety disorders, PTSD, adjustment disorder, anorexia nervosa, unspecified eating disorder, Tourette's Syndrome, ADHD, persistent tic disorder, enuresis)	K‐SADS‐PL	4 days (18 h)
				CY‐BOCS	
				GAD‐7	
				COIS‐R	
Stupar‐Rutenfrans et al. (2017), Netherlands	To increase our understanding of how mobile virtual reality exposure therapy (VRET) can help reduce speaking anxiety	35 university students (19–25 years)	Performance anxiety	PRCA‐24	5 min per video
				STAI	The average amount of practice for video 1 was three times (*M* = 3.03, SD = 1.04), for video 2 was four times (*M* = 4.00, SD = 0.87), and for video 3 was three times (*M* = 3.43, SD = 1.10).
Quasi‐experiments					
Finn et al. (2009), USA	To examine the role of brief repeated exposure in reducing public speaking state anxiety among students compared to a control condition	140 university students (*M* = 19.08, SD = 1.40, 18–29)	Performance anxiety	A‐STAI	1 session (15 min)
				PRCA‐24	
Franklin et al. (1998), USA	To determine whether a simplified version of CBT that omitted anxiety management training (AMT) and focused on ERP is effective with paediatric OCD comparing a weekly treatment condition with an intensive treatment condition	14 patients (*M* = 14.1, SD = 2.2, 10–17)	OCD	Y‐BOCS	For intensive treatment, mean of 18 sessions over 1 month (90 min per session). For weekly treatments, mean of 16 sessions over 4 months (60 min per session)
				Obsessive‐compulsive symptom ratings	
Hoffmann and Odendaal (2001), South Africa	To examine the effect of systematic desensitisation and instructional learning on the response patterns associated with dog phobia compared to a control condition	27 university students (experimental group: *n* = 12, *M* = 20.4, 18–28Control group: *n* = 15, *M* = 19.9, 18–25)	Specific or simple fears/phobia	BAT	5–7 weekly sessions (60 min per session)
				10‐Point self‐report anxiety scale	
				Plasma ACTH	
				Distance of dog approach	
Longo and Vom Saal (1984), USA	To evaluate the efficacy of graded imaginal exposure with or without deep breathing exercises for speech anxiety	60 university students (*M* = 20.9, 18–42)	Performance anxiety	Confidence inventory	3 sessions (30 min per session)
				Post‐speech questionnaire	
				Behavioral observation (timed behavioral checklist for performance anxiety)	
				GSR	
				HR	
Experiments					
Deacon et al. (2010), USA	To examine the effects of augmenting exposure therapy with the judicious use of safety behaviours	33 university students (*M* = 19.51, SD = 1.35, 18–23)	Specific or simple fears/phobia	BAT	1 session (30 min)
				CLQ	
				ASI‐3	
Dibbets et al. (2013), Netherlands	To investigate the effect of a retrieval cue on the return of spider fear comparing the same context to a new context	33 university students (*M* = 21.4)	Specific or simple fears/phobia	SCID‐I	2 session (116.38–152.38 min in total)
				SPQ	
				FSQ	
				BAT	
				SUD	
Goetz and Lee (2015), USA	To examine the extent to which different safety behaviours (i.e., no safety behaviours vs. restorative vs. preventive safety behaviors) enhanced or weakened symptoms of contamination fear during a single session of exposure	67 university students (*M* = 23.27, SD = 7.3)	Specific or simple fears/phobia	DASS	1 session (unspecified)
				OCI‐R	
				Anticipated fear	
Healey et al. (2017), UK	To determine if control (high or low) influenced the effectiveness of exposure therapy	96 university students (*M* = 22.2, SD = 5.9, 18–45)	Specific or simple fears/phobia	FSQ	1 session (1 h)
				FQ	
				GAD‐7	
				BAT	
				GSR	
Johnson and Casey (2015), USA	To test whether the reminder of a conditioned stimulus (cue) 10 min prior to extinction would block the recovery of fear memory	74 subjects (36 adults [*M* = 24.55], 38 adolescents [*M* = 14.65])	N/A	SCR	2 session (12 min 16 s per session)
Kim (2008), South Korea	To investigate the effects of two music therapy approaches, improvisation‐assisted desensitization and music‐assisted progressive muscle relaxation and imagery, on alleviating the symptoms of music performance anxiety among student pianists	30 subjects (*M* = 20)	Performance anxiety	Four types of visual analogues scales: Music performance anxiety, stress, tension, and comfort	6 weekly sessions (30 min per session)
				State portion of STAI	
				Music performance anxiety questionnaire	
Kircanski et al. (2012), USA	To examine whether the features of gradual, blocked exposure or random, variable exposure were necessary to achieve clinical improvement in contamination‐related fears	50 university students (*M* = 19.64, SD = 2.46)	Specific or simple fears/phobia	Y‐BOCS	3 sessions (total of 126 min)
				BAT	
				SUDS	
				HR	
				SCR	
Kondaš (1967), Czechoslovakia	To compare group desensitization, autogenic training (relaxation), gradual imaginal exposure without relaxation in children and in young adults	13 university students (*M* = 21.9), high school students (*M* = 13)	Performance anxiety	FSS	4 imaginal exposure sessions versus 5 systematic desensitisation sessions
Lent and Russell (1978), USA	To compare the efficacy of a combined desensitization‐study‐skills training strategy a cue‐controlled desensitization for the treatment of test anxiety (study‐skill training only (SS) versus systematic desensitization and study‐skills training (SD + SS) versus cue‐controlled desensitization and study‐skills training (CCD + SS) versus No treatment control condition (NTC))	66 university students (*M* = 20.6)	Performance anxiety	TAS	5 weekly sessions (60 min per session)
				AAT	
				AD	
				STAI‐T	
Müller et al. (2011), Germany	To compare the efficacy of a computer‐based one‐session treatment of exposure to spider pictures compared to neutral pictures in reducing fear and avoidance behaviour in spider‐fearful individuals	36 university students (*M* = 23.17, SD = 4.21, 18–34)	Specific or simple fears/phobia	FSQ	1 session (27 min)
				BAT	
Olatunji et al. (2012), USA	To investigate whether evoking the experience of disgust in blood injection injury phobia compared to a neutral condition may lead to less fear, disgust, and avoidance	44 university students (*M* = 18.93, SD = 0.97, 18–21)	Specific or simple fears/phobia	IPS‐anx	1 session (14 min video + BAT time unspecified)
				STAI‐T	
				BAT	
Olatunji et al. (2017), USA	To examine the effects of exposure in multiple contexts (compared with single context or control) on fear reduction and renewal and the moderating effect of baseline threat‐specific and non‐specific emotionality	108 university students (*M* = 19.22, SD = 1.19)	Specific or simple fears/phobia	Fear of snakes questionnaire	1 session (29 min + BAT time unspecified)
				ADIS‐IV phobia module	
				STAI‐T	
				BAT	
Pace‐Schott et al. (2012), USA	(1) to examine whether simulated exposure therapy in spider‐phobic subjects would lead to greater retention of fear extinction(2) to examine whether sleep would enhance generalization of the extinction memory to a novel spider	66 university students (*M* = 19.9, 18–28)	Specific or simple fears/phobia	FSQ	2 sessions (60 min per session)
				STAI‐T	
				STAI‐S	
				SCR	
				Electrocardiography	
				Electromyography	
Pace‐Schott et al. (2009), USA	To examine the effects of sleep on fear conditioning, extinction, extinction recall, and generalization of extinction recall in healthy humans	59 community participants (*M* = 23, SD = 4, 18–35)	N/A	SCR	‐‐
Possis et al. (2013), USA	To examine the relative efficacy of cognitive restructuring compared to behavioural experiments or psychoeducation conditions	61 university students (*M* = 20, SD = 3.36)	Social anxiety/phobia	ASI	1 session (5 min)
				SIAS	
Powers et al. (2008), USA	To examine the effect of attributional processes concerning medication‐taking on return of fear following exposure‐based treatment with participants allocated to one of these conditions: Waitlist, psychological placebo, exposure only, exposure plus inactive pill	95 community participants and university students (*M* = 20.11, SD = 6.23, 18–60)	Specific or simple fears/phobia	CIDI	1 session (30 min)
				BAT	
				CLQ	
Rachman et al. (2011), Canada	To assess the degree to which exposure while using safety behaviours is an effective intervention for contamination fears	80 university students (*M* = 20.62, SD = 3.30)	OCD	Ratings of contamination, fear, danger, and disgust	36 trials across 2 sessions
Rothbaum et al. (1995a), USA	To examine the efficacy of virtual reality graded exposure treatment for acrophobia compared to a waitlist condition	20 university students (*M* = 20, SD = 4)	Specific or simple fears/phobia	AQ	7 sessions (35–45 min per session)
				Rating of fear questionnaire	
				SUDS	
Scheveneels et al. (2017), Belgium	To examine the effect of providing information about the degree to which the extinction stimulus is typical (as opposed to unique/special) on the generalization of extinction to a new but similar stimulus	69 university students (*M* = 21.3, SD = 4.18)	N/A	GSR	‐‐
Shin and Newman (2018), USA	To examine the clinical utility of retrieval cues and completing the follow‐up in either the same or different context with an analogue sample of people with public speaking anxiety	65 university students (*M* = 19, SD = 2.05)	Performance anxiety	SPS	1 session (20 min)
				SSPS	
				BAT	
				SUDS	
				HR	
Tang et al. (2015), Taiwan	To assess the intervention effects of four‐session EMDR in reducing the severity of the psychological impact of Typhoon Morakot (including disaster‐related anxiety, general anxiety, and depression) in Taiwanese adolescents compared to treatment as usual (TAU)	83 high school students (EMDR: *n* = 41,*M* = 14.24 years old, SD = 0.99, 12–15; TAU: *n* = 42, *M* = 14.48 years old, SD = 0.92, 12–15)	PTSD (MDD)	The Taiwanese version of the multidimensional anxiety scale for children	3 sessions (30–40 min per session)
Vrielynck and Philippot (2009), Belgium	To investigate whether during imaginal exposure focusing on features that define the specificity of a stressful personal memory (i.e., the unique episodic procedure) leads to less distress than focusing on generic elements of such a memory (i.e., the generic procedure)	49 university students (*M* = 19.8, SD = 4.20, 17–46)	Social anxiety/phobia	STAI	2 sessions (15 min per session)
				SUDS	
Wolitzky and Telch (2009), USA	To investigate the efficacy of an exposure augmentation strategy in which the phobic individual is encouraged to enact actions that are in direct opposition to the fear action tendencies associated with acrophobia compared with exposure only, placebo control or waitlist control conditions	88 community members and university students (*M* = 20.08, 18–64)	Specific or simple fears/phobia	BAT	1 session of six 6‐min trials (36 min)
				Fear activation during exposure	
				Peak fear	
				HR	
				AQ	
Randomised controlled clinical trial					
Capaldi et al. (2016), USA	To examine the relationship between improvements in adolescent ratings of therapeutic alliance and reductions in PTSD severity over time among adolescent girls during prolonged exposure therapy for adolescents (PE‐A) versus client‐centred therapy (CCT), as well as to examine differences in changes in alliance between treatment groups	61 patients (*M* = 15.3, SD = 1.5, 13–18)	PTSD	CPSS	5–8 sessions (15–45 min)
				*Within group d = *2.54 (pre–post), large	
				*Between groups d* = 0.85(post), large	
Ertl et al. (2011), Uganda	To examine whether individual‐based, trauma‐focused narrative exposure therapy is feasible and effective in reducing PTSD symptoms, compared to an academic catch up program or a waitlist condition, in traumatized former child soldiers living in the Internally Displaced persons camps of Northern Uganda when carried out by trained local lay therapists directly in the communities	85 patients (*M* = 18)	PTSD	CAPS	8 sessions (90–120 min per session)
				*Within group d* = 1.18 (pre‐3M), large	
				*d* = 1.80 (pre to 12M FU), large	
				*Between groups*	
				*Compared to Active control d* = −0.05(3M^c^)	
				*d* = 0.41(12 FU), small to moderate	
				*Compared to waitlist d* = 0.31(3M), small	
				*d* = 0.45(12M FU), small to moderate	
Flack et al. (2018), Germany	To investigate the efficacy of interoceptive exposure as an adjunctive treatment, compared to relaxation, to enhance reductions in fear of pain in a sample of adolescents with chronic pain enrolled in intensive interdisciplinary pain treatment	110 patients (*M* = 14.4, SD = 1.7, 11–17)	Specific or simple fears/phobia (chronic pain)	GFOPQ‐C^f^	5 sessions (30 min per session)
				CASI	
				AQP	
				*Within group d* = 0.73 (pre–post), moderate to large	
				*d* = 1.05 (pre‐3M FU), large	
				*Between groups d* = 0.34(post), small	
				*d* = 0.23(3M FU), small	
Foa et al. (2013), USA	To compare a prolonged exposure program modified for adolescents with supportive counselling among adolescent girls with sexual abuse‐related PTSD	61 patient (*M* = 15.3)	PTSD	CPSS‐SR^f^	Up to 14 weekly sessions (60–90 min sessions)
				CGAS	
				K‐SADS	
				*Within group d* = 2.72 (pre‐post) large	
				*d* = 1.87 (pre‐12M FU), large	
				*Between‐groups d* = 1.01(post), large	
				*d* = 0.81(12M FU), large	
Gilboa‐Schechtman et al. (2010), Israel	To examine the efficacy and maintenance of developmentally adapted prolonged exposure (PE‐A) compared to active control time limited dynamic therapy (TLDP‐A) for reducing post‐traumatic and depressive symptoms in adolescent victims of single event traumas	38 patients (*M* = 14.05, SD = 2.27, 12–18)	PTSD (externalizing, internalising and combined disorders)	CPSS^f^	12–15 weekly sessions (60–90 min)
				CGAS	
				*Within group d* = 1.65 (pre‐post) large	
				*d* = 1.40 (pre‐17	
				M FU), large	
				*Between‐groups d* = 0.49(post), small to moderate	
				*d* = 0.22(17M FU), small to moderate	
Kaczkurkin et al. (2016), USA	To investigate whether high levels of anger resulted in poorer exposure treatment outcomes in a sample of female adolescents with PTSD while comparing a client‐centred therapy condition to PE‐A	61 patients (*M* = 15.3, SD = 1.52, 13–18)	PTSD	CPSS‐I^f^	8–14 weekly sessions (60–90 min per session)
				NMR	
				STAXI52	
				*Within group d* = 2.49 (pre‐post) large	
				*d* = 3.00 (pre‐12M FU), large	
				*Between‐groups d* = 0.83(post), large	
				*d* = 0.88(12M FU), large	
McLean et al. (2015), USA	To determine the i) relationship between changes in negative trauma‐related cognitions and changes in PTSD severity in adolescents receiving PTSD treatment with either client‐centred therapy or PR‐A; ii) relationship between changes in negative trauma‐related cognitions and changes in depressive symptoms during PE	61 patients (*M* = 15.3, SD = 1.5, 13–18)	PTSD	C‐PTAS	14 weekly sessions (60–90 min per session)
				CPSS‐I	
				Effect sizes reported in Foa et al., 2013 (above)^d^	
Neziroglu et al. (2000), USA	To explore if adding behaviour therapy to medication would enhance treatment efficacy for adolescents with OCD	10 patients (*M* = 14.5, SD = 2.4, 10–17)	OCD (ADHD, Trichotillomania)	CY‐BOCS^f^	20 weekly sessions (90 min per session)
				CGI‐I	
				CGI‐S	
				NIMH–GOCS	
				*Within group d* = 1.83 (pre‐post) large	
				*d* = 2.65 (pre‐FU^e^), large	
				*Between group d* = 0.24(post), small	
				*d* = 1.06(FU), large	
Rossouw et al. (2018), South Africa	To evaluate the comparative effectiveness of prolonged exposure and supportive counselling in adolescents with PTSD	63 patients (*M* = 15.35, 13–18)	PTSD	MINI‐KID	7–14 weekly (60‐min sessions)
				CPSS‐SR^f^	
				CGAS	
				*Within group d* = 3.81 (pre‐post) large	
				*d* = 4.38 (pre‐2 YR FU^e^), large	
				*Between group d* = 1.22(post), large	
				*d* = 1.02(2 YR FU), large	
Scheck et al. (1998), USA	To compare EMDR treated patients to active listening treated patients used diagnosed with PTSD or traumatised without PTSD to compare the effects of treatment	60 patients (*M* = 20.93, 16–25)	PTSD	STAI‐S^f^	2 sessions (90 min per session)
				Penn inventory for posttraumatic stress disorder	
				*Within group d* = 1.65 (pre‐post) large	
				*Between group d* = 0.66(post), moderate	
Zaboski et al. (2019), USA	To investigate whether exposure therapy compared to an active control condition would lead to greater changes in social anxiety, fear of negative evaluation and depression for college students	31 university students	Social anxiety/phobia (other anxiety disorders	LSAS	6 weekly sessions (2 h per session)
				SAQ‐A30^f^	
				BFNE	
				*Within group d* = 1.14 (pre‐post) large	
				*d* = 0.94(post), large	

Abbreviations: AAT, Achievement Anxiety Test; AD, Anxiety Differential; ADIS‐C, Anxiety Disorders Interview Schedule for DSM‐IV: Child Version; ADIS‐IV phobia module, ADIS‐IV specific phobia module from the Anxiety Disorders Interview Schedule for the DSM‐IV; ADIS‐P, The Anxiety Disorders Interview Schedule for Children‐Parent Version; AQ, Acrophobia Questionnaire; AQP, Anxiety Questionnaire for Pupils; A‐STAI, A‐State version of the STAI; ASI, Anxiety Sensitivity Index; BAT, Behavioural Avoidance/Approach Test; BFNE, Brief Fear of Negative Evaluation Scale; CASI, Child Anxiety Sensitivity Index; CGAS, Children's Global Assessment Scale; CGI‐S, Clinical Global Impressions‐Severity of Illness Scale; CGI‐I, Clinical Global Impressions‐Improvement Scale; CIDI, Composite International Diagnostic Interview; CIS‐C/P, Columbia Impairment Scale‐Parent & Child versions; COIS‐R, Child OCD Impact Scale‚ Revised; C‐PTAS, Child Post‐Trauma Attitudes Scale; CLQ, Claustrophobia Questionnaire; CPSS‐SR, Child PTSD Symptom Scale; Self‐Report; CPSS‐I, Child PTSD Symptom Scale‐Interview; CY‐BOCS, Children's Yale‐Brown Obsessive Compulsive Scale; CY‐BOCS‐CA‐SR, Children's Yale‐Brown Obsessive Compulsive Scale Child/Adolescent Self‐Report Symptom Checklist; DASS, Depression Anxiety and Stress Scale; DOCS, Dimensional Obsessive Compulsive Scale; EMDR, Eye Movement Desensitization and Reprocessing Therapy; ERP, Exposure and Response Prevention; FQ, Fear Questionnaire; FSS, Fear Survey Schedule; FSQ, Fear of Spiders Questionnaire; GAD‐7, Generalised Anxiety Disorder 7 item measure; GFOPQ‐C, German version of the Fear of Pain Questionnaire for Children; GSR, Galvanic Skin Response; HR, Heart Rate; IPS‐Anx, Injection Phobia Scale‐Anxiety; K‐SADS‐PL, Kiddie‐Schedule for Affective Disorders and Schizophrenia for School‐Age Children‚ Present and Lifetime Version; LSAS, Liebowitz Social Anxiety Scale Self Report Measure; LSAS‐CA, Liebowitz Social Anxiety Scale for Children/Adolescents; MASC, Multidimensional Anxiety Scale for Children; MINI‐KID, Mini International Neuropsychiatric Interview for Children and Adolescents; NIMH–GOCS & CGI, National Institute of Mental Health Global Obsessive‐Compulsive Scale; NMR, Negative Mood Regulation Scale; OCI‐R, Obsessive‐Compulsive Inventory‐Revised; PE, Prolonged Exposure; PRCA‐24, Personal Report of Communication Apprehension scale; PRCS, Personal Report of Confidence as a Speaker; PSAS, Public Speaking Anxiety Scale; PTSD, Post‐Traumatic Stress Disorder; SAQ‐A30, The Social Anxiety Questionnaire for Adults; SCAS, Spence Children Anxiety Scale; SCARED, Screen for Child Anxiety Related Disorders; SCID‐I, Structured Clinical Interview for DSM‐IV Axis I; SCR, Skin Conductance Response; SIAS, Social Interaction Anxiety Scale; SPQ, Spider Phobia Questionnaire; SPS, Social Phobia Scale; SSPS, Self‐Statements during Public Speaking Scale; STAI, State‐Trait Anxiety Inventory; STAI‐S, State‐Trait Anxiety Inventory; State Form; STAI‐T, State‐Trait Anxiety Inventory; Trait Form; STAXI‐2, Spielberger State‐Trait Anger Expression Inventory‐2; SUDS, Subjective Units of Discomfort/Distress Scale; TAS, Test Anxiety Scale; UCLA PTSD‐RI, University of California at Los Angeles PTSD Reaction Index; VOCI, Vancouver Obsessive Compulsive Inventory; VOCI‐MC, Vancouver Obsessive Compulsive Inventory‐Mental Contamination; Y‐BOCS, Yale‐Brown Obsessive Compulsive Scale.

^a^ Psychoeducation about exposure therapy and exposure treatment planning (e.g., generating fear hierarchy) were included in the total number of exposure sessions.

^b^ For the case series studies, only participants/patients within the relevant age ranges 13.5 and 24.4 were reported in the table.

^c^ Included as a post effect size for the purposes of average effect sizes.

^d^ Effect size not included for the purposes of calculating average effect sizes.

^e^ 52 weeks follow‐up data used to calculate effect size.

^f^ Effect size based on this measure.

**TABLE 4:** Characteristics of exposure and optimisation strategies

		In vivo	Imaginal	Graded	Flooding	Response prevention	With eye movement (EMDR)	Relaxation/Breathing exercises	Psychoeducation	Cognitive strategies	Intensive	Technology‐assisted	Other	
Case reports	Abramowitz et al., 2002	✓	✓	✓		✓			✓		✓			
	Buchanan & Houlihan, 2008	✓		✓				✓						
	Chok et al., 2010	✓		✓									✓	Reinforcements (social praise)
	Frye & Spates, 2012	✓	✓	✓				✓	✓		✓	✓	✓	Interoceptive exposure, mindfulness, emotional regulations skills
	Kitchiner, 2000		✓				✓							
	Rothbaum et al., 1995b		✓	✓								✓		
	Newman & Adams, 2004	✓		✓				✓					✓	Modelling (from mother)
	Saigh, 1987		✓		✓			✓						
	Whiteside & Abramowitz, 2006	✓	✓	✓		✓			✓		✓			
	Woods et al., 2000	✓		✓		✓								
	Wu & Storch, 2016	✓	✓	✓		✓			✓	✓			✓	Family involvement
Case series	Burton et al., 2017	✓		✓									✓	Reinforced practice & therapist modelling
	Culver et al., 2012	✓										✓		
	Farrell et al., 2016	✓				✓			✓	✓	✓		✓	Parental training
	Hendriks et al., 2017	✓	✓	✓				✓	✓		✓		✓	Parental training
	Iniesta‐Sepulveda et al., 2018	✓		✓		✓		✓	✓				✓	Parental training
	Marr, 2012		✓				✓							
	Whiteside et al., 2008	✓	✓	✓		✓			✓	✓	✓		✓	Parental training
Cohort studies	A. Baker et al., 2010	✓												
	Fischer et al., 1998	✓				✓			✓				✓	Group‐based
	Kahlon et al., 2019		✓	✓					✓			✓		
	Krebs et al., 2015	✓	✓	✓		✓			✓				✓	With medication
	Mathes et al., 2019	✓	✓			✓								
	Morina et al., 2015		✓									✓		
	Riise et al., 2018	✓				✓			✓				✓	Parental training
	Stupar‐Rutenfrans et al., 2017		✓	✓								✓		
Experiments	Deacon et al., 2010	✓		✓					✓					
	Dibbets et al., 2013	✓		✓										
	Goetz & Lee, 2015	✓		✓										
	Healey et al., 2017		✓									✓		
	Johnson & Casey, 2015												✓	Fear conditioning & extinction
	Kim, 2008		✓	✓				✓						
	Kircanski et al., 2012	✓		✓									✓	Graded or random
	Kondaš, 1967		✓	✓				✓						
	Lent & Russell, 1978		✓	✓				✓						
	Müller et al., 2011		✓									✓		
	Olatunji et al., 2012	✓	✓	✓								✓		
	Olatunji et al., 2017	✓	✓	✓								✓		
	Pace‐Schott et al., 2012		✓									✓		
	Pace‐Schott et al., 2009												✓	Fear conditioning and extinction
	Possis et al., 2013	✓							✓					
	Powers et al., 2008	✓												
	Rachman et al., 2011	✓			✓	✓							✓	Safety behaviours versus no safety behaviours
	Rothbaum et al., 1995a		✓	✓								✓		
	Scheveneels et al., 2017												✓	Fear conditioning and extinction
	Shin & Newman, 2018	✓												
	Tang et al., 2015		✓				✓		✓					
	Vrielynck & Philippot, 2009		✓					✓						
	Wolitzky & Telch, 2009	✓		✓									✓	With or without oppositional actions
Quasi‐experiments	Finn et al., 2009	✓												
	Franklin et al., 1998	✓	✓	✓		✓			✓					
	Hoffmann & Odendaal, 2001	✓		✓				✓						
	Longo & Vom Saal, 1984		✓	✓				✓						
Randomised controlled clinical trials	Capaldi et al., 2016	✓	✓	✓				✓	✓					
	Ertl et al., 2011		✓						✓					
	Flack et al., 2018		✓	✓						✓			✓	Tapping and imagery techniques
	Foa et al., 2013	✓	✓	✓				✓	✓					
	Gilboa‐Schechtman et al., 2010	✓	✓	✓				✓	✓					
	Kaczkurkin et al., 2016	✓	✓	✓				✓	✓					
	McLean et al., 2015	✓	✓	✓				✓	✓					
	Neziroglu et al., 2000	✓		✓		✓							✓	Fluvoxamine medication
	Rossouw et al., 2018	✓	✓	✓				✓	✓					
	Scheck et al., 1998		✓				✓	✓						
	Zaboski et al., 2019	✓		✓		✓			✓					

**FIGURE 2:**
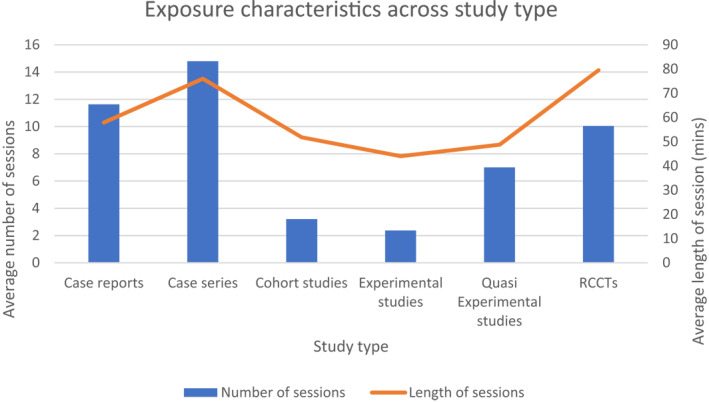
Exposure characteristics across study type

Figure 2 provides a summary of the length and number of sessions across each study type. Typically, exposure sessions were fewer and shorter in the experimental studies, with the longest exposure sessions occurring in the randomised clinical trials and the greatest number of sessions occurring in the case series. We will provide a review of our main areas of focus: studies evaluating the efficacy of exposure, and studies identifying moderators of exposure. The studies will be organised according to the type of study. Within each section, we will first review the evidence from RCCTs, followed by experimental studies, quasi‐experimental studies, cohort studies, case series and case studies.

### Efficacy of exposure

#### Randomised controlled clinical trials (RCCT)

Eleven RCCTs directly examined the efficacy of exposure among 14–24‐year‐olds. All studies provided a comparison of exposure treatment compared to an active control condition, providing a conservative test of efficacy. Notably, no studies focused on youth with diagnosed DSM‐5 anxiety disorders and instead focused on OCD, PTSD and subclinical presentations of anxiety. In many cases, the administration of exposure was integrated with other intervention strategies (e.g., relaxation/breathing exercises, psychoeducation, cognitive strategies, and eye movement desensitisation and reprocessing (EMDR)), preventing conclusions about the unique effects of exposure delivered as an ingredient on its own. If within and between group effect sizes were not provided they were calculated using www.psychometrica.dl/effect_sizes.html (See Table 3). The average within‐subject effect size for exposure therapies was very large at post‐treatment (*d* = 1.97) and follow‐up (*d* = 2.31). This means that overall there was a substantial change in anxiety/fear symptoms immediately following exposure‐based therapies. This change appears to be maintained in the short to medium term. The average between‐group effect sizes comparing exposure to an active control that did not specifically include exposure were moderate to large at post‐treatment (*d* = 0.65) and follow‐up (*d* = 0.66). This means that compared to other treatments for anxiety and related disorders, exposure‐based therapies are superior (See Table 3 for more information).

Of the 11 RCCTs, eight included exposure in the treatment of PTSD with large within group effect sizes (Post‐treatment: *d* = 2.29; Follow‐up: *d* = 2.49) as well as moderate to large average between group effect sizes when compared to active controls (Post‐treatment: *d* = 0.72; Follow‐up: *d* = 0.67). These studies all administered imaginal exposure delivered in combination with graded, in vivo exposure and relaxation/breathing exercises (e.g., Gilboa‐Schechtman et al., 2010) or through narrative exposure therapy (Ertl et al., 2011), or in combination with EMDR (Scheck et al., 1998). All of these approaches were found to be more efficacious in reducing PTSD severity scores or PTSD diagnoses in adolescents and young adults with PTSD when compared to an active control condition such as an academic tutoring program (Ertl et al., 2011), active listening (Scheck et al., 1998), or dynamic therapy (Gilboa‐Schechtman et al., 2010). In a secondary analysis of another included trial (Foa et al., 2013), and one of the only studies to examine possible mechanisms of change, McLean et al. (2015) found that changes in negative trauma‐related cognitions resulting from exposure therapy mediated changes in PTSD symptoms.

The remaining three RCCTs focused on treatment of social anxiety symptoms (Zaboski et al., 2019), fear of pain (Flack et al., 2018), and OCD (Neziroglu et al., 2000). Both Zaboski et al. (2019) and Neziroglu et al. (2000) reported large effect sizes for exposure treatment conditions compared to an active control condition at post‐treatment and follow‐up assessments (See Table 3). For example, combining medication with graded, in vivo exposure with response prevention was more effective than medication alone in reducing OCD symptoms in adolescents with OCD (Neziroglu et al., 2000). Similarly, exposure significantly reduced social anxiety symptoms in university students (using group therapy with graded, in vivo exposure and response prevention) compared to a psychoeducation control (Zaboski et al., 2019). In contrast to these two studies observing large effect sizes, Flack et al. (2018) reported small, non‐significant effect sizes at both post‐treatment and follow‐up favouring interoceptive exposure over relaxation for reducing fear of pain in a clinical sample of adolescents with chronic pain (Flack et al., 2018).

#### Experimental studies

Twenty‐three experimental studies examined the efficacy of exposure, with most studies examining the effects on specific fears or phobias (*n* = 12; 52%) and in samples of university students (*n* = 19; 83%). There were few experimental findings where strong conclusions about the efficacy of exposure could be drawn, due to considerable variations in aims, limited methods (e.g., no random assignment) and lack of replication. Further, many of the studies were designed to test features which may facilitate exposure (these findings are reviewed in the next section).

Six of the experimental studies provided some information regarding the overall efficacy of exposure. In a sample of university students, both imaginal exposure with systematic desensitisation (which involved inducing deep muscle relaxation during imaginal graded exposure) and imaginal exposure with cue‐controlled desensitisation (modified systematic desensitisation with self‐administered cue‐controlled relaxation) significantly reduced performance anxiety, compared to study skills alone (Lent & Russell, 1978). In this study, neither exposure condition differed from each other in terms of outcomes. Following an intervention involving imaginal exposure and EMDR in a sample of high‐school students with PTSD, Tang et al. (2015) showed significantly lower anxiety compared to a treatment as usual condition. There was also preliminary evidence for the efficacy of group systematic desensitisation, in a small sample of children (11–15 years old, *n* = 23) and university students (mean age 21.9 years *n* = 13) showing significantly decreased performance anxiety compared to autogenic training (involving exercises of relaxed breathing, progressive muscle relaxation) and imaginal exposure alone (Kondaš, 1967).

In vivo exposure was found to be associated with reductions in anxiety/fear in several experimental studies using university students. In a single session, in vivo exposure was associated with significantly lower spider fear and avoidance than viewing neutral images (Müller et al., 2011). In vivo exposure was also associated with lower avoidance and anxiety for young people with a diagnosis of Specific Phobia (heights) when delivered weekly using virtual reality (VR) compared to a waitlist condition (Rothbaum et al., 1995a). Blocked and constant exposure (exposure that focused on repeating one step in each session using a gradual hierarchy) did not differ significantly from random and variable exposure in reducing contamination‐related fears for university students, immediately and 2 weeks later (Kircanski et al., 2012). This preliminary evidence suggests that in vivo exposure is effective in treating simple/specific fears and that the delivery may not necessarily need to be gradual or use a typical exposure hierarchy.

Two small experiments found exposure treatments to be less or no more efficacious than an alternative treatment (Kim, 2008; Possis et al., 2013). In university students with elevated social anxiety (*n* = 61), Possis et al. (2013) found that compared to a psychoeducation control, one session of cognitive restructuring (without exposure) was more efficacious at reducing social anxiety than one behavioural experiment (involving exposure). Further, there was no significant difference between improvisation‐assisted desensitisation (involving graded imaginal exposure) or music‐assisted progressive muscle relaxation (6 weekly sessions of 30 min) in reducing performance anxiety in young pianists (*n* = 30; Kim, 2008).

#### Quasi‐experimental studies

There were four quasi‐experimental studies identified in this scoping review, with three out of four studies testing exposure in university students with performance or specific fears and one clinical study of patients with OCD. All four studies showed significant reductions in anxiety symptoms following exposure. For example, in vivo exposure was effective in significantly reducing performance anxiety compared to students allocated to a control condition involving written assignments (Finn et al., 2009). Similarly, Longo and Vom Saal (1984) showed that graded imaginal exposure with or without breathing exercises showed greater reductions in performance anxiety in university students compared to a waitlist condition. In a third study, university students who reported high dog fears also demonstrated reductions in fears following exposure but still had significantly higher fear than a low dog fear control group (Hoffmann & Odendaal, 2001). Finally in an open clinical trial with a sample of children and adolescents with a diagnosis of OCD, exposure in either intensive or weekly treatment was associated with a significant reduction in OCD severity (Franklin et al., 1998).

*Cohort studies.* The scoping review identified eight cohort studies, one of which provided information about moderators of efficacy and is discussed in the next section. Three cohort studies provided information on the potential benefits of using virtual reality to deliver exposure to treat social/performance anxiety (Kahlon et al., 2019; Morina et al., 2015; Stupar‐Rutenfrans et al., 2017). Two cohort studies examined the efficacy of exposure in clinical adolescent populations with OCD (Fischer et al., 1998; Riise et al., 2018). Significant reductions in OCD symptoms were observed following group graded in vivo exposure (with response prevention) delivered weekly (Fischer et al., 1998) or intensively (Riise et al., 2018). Finally, two additional cohort studies of university students with contamination fears (Mathes et al., 2019) and fears of water (A. Baker et al., 2010), showed significant reduction in fears following the delivery of exposure treatments.

*Case series and case studies.* 11 case reports and seven case series reported favourable effects when using exposure to treat specific fears/phobias (Buchanan & Houlihan, 2008; Burton et al., 2017; Chok et al., 2010; Newman & Adams, 2004; Rothbaum et al., 1995b), performance anxiety (Culver et al., 2012), OCD (Abramowitz, 2002; Farrell et al., 2016; Iniesta‐Sepúlveda et al., 2018; Marr, 2012; Whiteside & Abramowitz, 2006; Whiteside et al., 2008; Woods et al., 2000; Wu & Storch, 2016), and PTSD (Frye & Spates, 2012; Hendriks et al., 2017; Kitchiner, 2000; Saigh, 1987). Of these studies, eight (44%) included youth under 18 years, 14 case studies/series (78%) employed in vivo exposure, and ten (56%) used imaginal exposure. All case studies/series reported favourable results in terms of reduction in symptoms or improvements in the percentage of young people achieving remission. For example, Hendriks et al. (2017) found that intensive prolonged exposure significantly reduced PTSD symptoms within 1 week without any non‐trauma focused techniques.

### Moderators of outcome

Of all the RCCTs and case studies reported above, none examined factors affecting the efficacy of exposure in young people. The evidence of moderators of outcome came from experimental, quasi‐experimental and cohort studies only. Twenty‐two studies, primarily using experimental approaches, tested a range of optimisation strategies among young people. The findings were preliminary in nature and require replication in clinical samples of young people given most studies (*n* = 20; 91%) were conducted with university student samples. Except for two studies on the timing of exposure relative to sleep (Pace‐Schott et al., 2009, 2012), the findings from the remaining 20 studies have not been replicated. The preliminary data on optimisation strategies should be considered with these limitations in mind.

*Focusing on specific details.* Using a randomised design, asking university students (*n* = 49) with elevated social anxiety to focus on the distinctive features of the stressful personal experience during imaginal exposure (e.g., participants were instructed to recall all the specific features of the target event, in detail) was significantly more effective at decreasing distress than focusing on generic elements (Vrielynck & Philippot, 2009).

*Challenging exposures*. The findings of one randomised controlled experiment of university students with height fears indicated that adding actions to make exposure more challenging (such as running towards the railing) was significantly more effective than exposure that did not include this feature in reducing reported fear (Wolitzky & Telch, 2009).

*Inadequate us*e. In adolescents with OCD who had received a non‐effective course of CBT and medication, Krebs et al. (2015) showed that 95.5% of the previous courses of CBT were rated as inadequate, primarily because there had not been a sufficient focus on exposure in the treatment.

*Multiple contexts.* Olatunji (2017) conducted a randomised controlled experiment in a community sample of young adults with Specific Phobia (Snakes; *n* = 108). Exposure delivered in multiple contexts resulted in significantly better outcomes in terms of avoidance and fear than when young people were exposed to videos of the feared object in single contexts.

*Evoking disgust*: Olatunji et al., 2012 showed that activating emotions of disgust prior to repeated exposure did not significantly enhance reduction of fear, avoidance or disgust in a university sample of students (*n* = 44) with high injection fears.

*Drug enhancement*. Powers et al. (2008) randomly allocated university students and community volunteers (*n* = 95) with elevated fears of enclosed spaces to take either yohimbine hydrochloride (a selective competitive alpha2‐adrenergic receptor antagonist that appears to enhance extinction learning) or placebo prior to exposure. Although there were no immediate differences in reduction of fear, with both groups showing significant fear reduction, participants in the drug condition were significantly less likely to experience a return of fear at the 1‐week follow‐up assessment.

*Safety behaviours.* Three randomised controlled experiments investigated the impact of safety behaviours on the efficacy of exposures in high fearful university samples. In a non‐clinical sample, Goetz and Lee (2015) found that allowing safety behaviours (e.g., hygienic wipes) after exposures to treat contamination fears was associated with significantly more rapid reductions in fear and behavioural avoidance compared to participants using safety behaviours prior to the exposure. Rachman et al. (2011) showed that safety behaviours used after exposure for contamination fears did not lead to significantly enhanced outcomes compared to when participants were not given the opportunity to use safety behaviours. Similarly, there was no significant difference in outcomes for participants with high claustrophobic fear when given access to safety aids during exposure compared to exposure without safety aids (Deacon et al., 2010).

*Cue reminders.* Four experimental studies have examined the impact of providing cues during the exposure process (as a reminder of an object/element associated with the feared event). For example, one randomised controlled experiment on university students with elevated performance anxiety conducted by Shin and Newman (2018) presented all participants with specific cues during exposure sessions and then randomly allocated participants to receive or not receive the cues during a behavioural avoidance test 1 week later. The reminder cues prevented a return of fear on two of three outcomes. In contrast, in another pseudo‐randomised experiment of university students with high spider fears, reminders of cues presented during the exposure did not significantly attenuate fear renewal in a different exposure context (Dibbets et al., 2013).

*Stimuli Information.* Two studies examining different types of information provision were included in the review. The first study examined safety information and the second study examined the degree to which the participant was told the extinction stimuli were like other stimuli. First, Johnson and Casey (2015) found that safety information presented 10 min prior to extinction attenuated the recovery of fear in both teenagers (*n* = 36) and young adults (*n* = 38). Participants who were not provided with safety information (during post retrieval extinction) displayed a robust recovery of the fear compared to those participants who were given the safety information. In the second experiment with university students (*n* = 69), Scheveneels et al. (2017) found that providing information about the object (artificial animal like objects) as ‘typical’ of feared stimuli promoted significantly greater generalisation of extinction, measured by lower shock expectancies, compared to atypical explanations.

*Timing*. In two pseudo‐randomised fear extinction experiments, conducting exposure sessions with young adults closer to sleep (i.e., in the evening as opposed to the morning) enhanced its efficacy, as demonstrated by physiological and subjective measures of negative affect, suggesting that sleep may facilitate memory reconsolidation (Pace‐Schott et al., 2009, 2012). First, Pace‐Schott and colleagues used a fear conditioning and extinction paradigm in two groups of young adults from the community: one group who were given 12 h of normal daytime waking after conditioning and extinction, and one group who was allowed a normal night's sleep. Using assessment of skin conductance response 12 h later, the Sleep group had significantly lower reaction (indicating less fear) to an unextinguished yet similar conditioned stimulus. In the second study, Pace‐Schott et al. (2012) conducted imaginal exposures with high spider fear university students, timing treatment either in the morning or in the evening. The group who was able to sleep after the exposure session showed less fearful responses (both subjective and physiological) during testing 12 h later.

*Control.* Healey et al. (2017) allocated high spider fearful young adults to one of two conditions: either high or low control over a joystick which could allow the digitally presented images of the feared object (spiders) to come closer or move further away. Although there were no significant differences between the groups in terms of arousal or subjective distress, those with greater control were significantly more successful in approaching a spider at the end of the session and less avoidant at follow‐up.

*Relaxation.* Few experimental studies that examined the effect of relaxation techniques on exposure outcomes. A quasi‐experiment with university students with elevated speech anxiety found that incorporating breathing exercises with imaginal exposure was significantly more effective than exposure without breathing exercises in reducing self‐reported and observed (but not physiological) measures of speech anxiety (Longo & Vom Saal, 1984). Similarly, one case study of a young adult with PTSD reported that incorporating mindfulness exercises before and after exposure was helpful as measured by verbal client feedback and overall reductions in self‐report measures of fear and anxiety (Frye & Spates, 2012).

### Stakeholder engagement

*Efficacy.* Stakeholders were unsurprised by the evidence supporting the efficacy of exposure, as those who had experience with the technique had found it helpful. One clinician believed that anxiety was secondary to other underlying issues so was trained not to target the anxiety specifically and hence had not used the technique. At the second stakeholder meeting, this same clinician had begun adopting the technique successfully.

*Moderators*. Based on their own experience, two of the moderators identified in the review were identified as important by stakeholders: timing of the exposures and control. Both youth and carers believed that exposure was effective when it was graded with “very small clear steps” that enabled the young person to feel “in control.” Both groups also voiced the importance of youth autonomy. Second, the review suggested that exposures closer to the time a young person goes to sleep may be more effective, yet in contrast, one youth found that engaging in exposures in the mornings was more helpful, because if she had exposure sessions in the afternoon she would spend the day catastrophising about it.

The remaining moderators identified in the review were not considered to be as important to stakeholders. However, stakeholders highlighted several other factors that they believed were important to the delivery and efficacy of exposure. For example, clinicians believed exposure was successful when youth had external support (e.g., from family or friends), felt understood, comprehended the treatment rationale, were willing and motivated to engage in treatment, and trusted the therapist.

Youth and carers identified the importance of carers being given clearly defined roles in the exposure process. Youth and carers often felt unsupported, with carers being unsure of their role in homework tasks and how much assistance to provide. Both groups also talked about the importance of therapeutic alliance (with outcomes from exposure being more favourable when the therapist was trusted and there was a positive relationship). Parents and carers emphasised the importance of using empathetic language, psychoeducation, rewards, preventing unhelpful reassurance giving, and having more than one family member or having friends engaged in supporting the young person.

Some of the strategies young people employed to help with exposure practice included realistic thinking, breathing exercises, self‐talk strategies, and mindfulness. Young people reported engaging in exposure exercises with a therapist or informally on their own or with peers. One young person received CBT strategies as a child and so found it “childish” to return to the same program again as an adolescent.

### Barriers to delivery

Many clinicians expressed difficulties motivating clients to engage in exposure and reported anxieties about their client's wellbeing which often led them to avoid using exposure despite its potential benefits. Clinicians emphasised that preserving the relationship with the client was a very important factor for them in deciding whether to conduct exposure and they often avoided implementing the strategy with teenagers due to fear of jeopardising the client relationship and engagement. For some clinicians, their own anxiety about and lack of confidence in conducting exposure impeded exposure delivery.

Both parent and youth stakeholder groups expressed reluctance to engage in group exposure therapy, perceiving it to be less effective than one‐on‐one exposure sessions as sessions were not personalised. While shared experiences may enhance group therapy, the clinicians expressed how it could be “very overwhelming for both clients and therapists” and difficult to personalise treatment.

The primary barrier to exposure faced by the parents was difficulty motivating the young person to take ownership of their treatment and complete homework exercises. A common factor that was brought up by carers was the difficulty they experienced supporting the young person through the exposure homework. Moreover, parents voiced feeling unsupported and untrained in helping their child engage in exposures. Although parents were more involved with treatment when their child was younger, as the children got older young people reported they typically wanted their parents to be less directly involved with treatment. Finally, one young person highlighted that what may seem to help in the short‐term may not translate to long‐term benefits: “in the moment I found [distraction] really beneficial, cause I'm like, well, I'm not really having to deal with it. Long‐term that was not beneficial at all.”

## DISCUSSION

This scoping review identified the nature and extent of published research evaluating exposure as an intervention in young people aged 14–24 years, a developmental period of particularly high risk for anxiety and related disorders. The review also highlighted the body of work that has examined factors that may affect the efficacy of and application of exposure. The review was informed by input from clinicians, parents/carers, and young people with lived experience of anxiety problems, describing their attitudes towards and experiences with exposure. Overall, the main conclusion is that there is a lack of clear, replicated evidence evaluating the efficacy of exposure among young people in the 14‐to‐24‐year age group, experiencing DSM5 anxiety disorders. The evidence from randomised clinical controlled trials is primarily focused on DSM‐IV anxiety disorders PTSD and OCD (with only one RCCT on elevated social anxiety symptoms) however notably these showed large effects and stronger outcomes compared to other active treatments that did not involve exposure. Evidence from other study designs also suggested that exposure, delivered either imaginally or in vivo, resulted in positive effects for reducing symptoms of OCD as well as Specific Phobias/fears and performance anxiety in this age group.

One of the substantial limitations of this body of work is that little can be concluded about the specific outcomes from and moderators of exposure. Although some experimental studies enabled the unique effects of a laboratory equivalent of exposure to be examined, there were no studies of this nature in clinical populations. Indeed, despite us only including treatment studies where exposure was the key treatment ingredient and was introduced from the start of treatment, exposure was rarely delivered on its own and was typically accompanied by other anxiety management techniques. This is an important limitation as the addition of these techniques may have positive or negative effects. For example, a recent meta‐analysis examining CBT for a younger cohort (5–15 years) identified stronger intervention effects when CBT with exposure was delivered *without* the addition of relaxation (Whiteside et al., 2020). Furthermore, while a broad range of optimisation strategies have been evaluated within the identified body of research, the timing of exposures relative to going to sleep (where an exposure immediately prior to sleep appeared to augment the reduction of spider fears), was the only one that had been replicated in a second study. Together these issues highlight the need for a systematic program of robust and reproducible research that examines the efficacy of exposure and the impact of additional anxiety management strategies (such as relaxation, cognitive restructuring) on the efficacy related to exposure in 14–24‐year‐olds is an essential next step for research.

A further limitation of the available literature is the extent to which it reflects stakeholders' experiences and preferences. Stakeholder discussions highlighted the importance of a number of potential optimisation strategies that have yet to be evaluated in this population such as the benefits of informal exposures with peers, completion of out of session exposures (homework), and parental role definition and support. These provide clear directions for future research. There were a couple of notable exceptions and these present interesting dilemmas for clinical practice given the potential mismatch between expectations or experiences and research evidence. Clinicians reported sometimes being reluctant to engage in exposure due to the risk that it might rupture the therapeutic relationship, however, in one randomised controlled trial, client‐reported therapeutic alliance was stronger in exposure treatments for PTSD compared to client centred therapy (Capaldi et al., 2016). Contrary to some clinicians' expectations, this preliminary evidence suggests there is potential for exposure to augment rather than disrupt the therapeutic alliance. This suggestion is consistent with findings from a recent meta‐analysis that attrition from exposure with response prevention is generally lower than from other therapies among youth (under 17‐year‐olds) with OCD (Johnco et al., 2020). Finally, stakeholders highlighted that a graded approach is important to promote engagement and a sense of control among young people. While this has not been directly tested, Kircanski et al. (2012) showed in an experimental study that a graded approach to exposure delivered similar outcomes to exposures of random difficulty among young people with OCD. Whether the graded approach increases young people's readiness to engage in exposure within clinical settings remains to be seen. Indeed, the stakeholder discussions emphasised the need for more developmentally sensitive research that identifies how best to help young people, parents and clinicians overcome concerns about conducting exposure within treatment.

It was clear from our stakeholder groups that exposure was not always offered to young people seeking help for anxiety problems or not always delivered in an adequate manner. In support of this, one cohort study in our review found that client experiences of previous CBT were inadequate at treating OCD due to insufficient use of exposure (Krebs et al., 2015). The authors suggested that clinicians may avoid behavioural aspects of the treatment due to fear of triggering distress in the client. As mentioned, this idea was supported by our clinician stakeholders, some of whom expressed reluctance to use exposure with their clients. This is also consistent with several studies in younger cohorts showing limited use of exposure in the community (Higa‐McMillian et al., 2017; Whiteside et al., 2016).

Further critical questions that remain unanswered by the existing research evidence about exposure for the treatment of anxiety disorders in young people include how outcomes and moderators differ across adolescence. Indications from animal studies that fear learning and extinction may differ during different periods of adolescence (K. D. Baker et al., 2014) highlight the importance of future studies taking a developmental perspective. The question of how well exposure works and how it might be optimised for specific anxiety disorders also remains unknown‐with most clinical studies conducted with young people with OCD or PTSD. Furthermore, where there is evidence of the efficacy of exposure there has been very little evaluation of the mechanisms of change.

Consistent with a scoping review approach (Tricco et al., 2018), methodological quality was not appraised and, given the wide variation in study research questions and approaches, we took a descriptive approach to clarifying the identified research. We hope that with a growing evidence‐base future systematic reviews will include both meta‐analytic approaches to synthesise findings and systematic appraisal of research quality.

## CONCLUSION

Exposure is typically seen as the central component in anxiety treatments for young people. Yet there are fundamental and substantial gaps in our knowledge of exposure‐based therapies in treating anxiety‐related conditions specifically in adolescents. Few robust conclusions can be drawn about the outcomes from exposure and potential ways to optimise it given the lack of replicated research using rigorous methods. It is unclear whether exposure is effective as a strategy on its own and whether adjunctive anxiety management strategies help or hinder. Given the high prevalence of anxiety disorders among adolescents and the long‐term consequences of ineffective treatment, it is paramount for a systematic program of high‐quality research to be undertaken to address the fundamental gaps identified in this review to make clinical recommendations that are developmentally sensitive to the needs of adolescents and their families.

## AUTHOR CONTRIBUTIONS

Co‐first authors Alessandra Teunisse, Lorna Pembroke: AT and LP contributed equally to this manuscript; data curation, formal analysis, project administration, writing‐original draft, writing‐review & editing. Jennie Hudson: conceptualisation, funding acquisition, methodology, project administration, supervision, writing‐review & editing. Ron Rapee, Viviana Wuthrich and Cathy Creswell: conceptualisation, funding acquisition, methodology, writing‐review & editing. Maddison O‐Gradey Lee: conceptualisation, funding acquisition, methodology, data curation, writing‐review & editing. Megan Sy: Data curation, writing‐review & editing.

## CONFLICTS OF INTEREST

The authors have declared that they have no competing or potential conflicts of interest.

## ETHICS CONSIDERATIONS

Formal ethical approval was not required as this study used data from published studies.

## Supporting information

Supporting Information S1

## Data Availability

The study used data extracted from published studies. Data used in the study is available on request from Jennie Hudson.
