# Supplementary material for: A scoping review investigating the use of exposure for the treatment and targeted prevention of anxiety and related disorders in young people
Source: JCPP Adv. 2022 May 20;2(2):e12080. doi: 10.1002/jcv2.12080 (PMC10242975; doi:10.1002/jcv2.12080)
Supplement: Supplementary file 1 — Supporting Information S1 [file JCV2-2-e12080-s001.docx]

**Appendix S1.**

**Coding of Exposure characteristics and optimisation strategies (See Table 4)**

‘In vivo’ exposure involves directly facing a feared situation or object in real life. For example, touching a spider, speaking in front of the class, visiting the scene of the trauma.

‘Imaginal’ exposure involves vividly imagining a feared situation or object. This type of exposure can also involve the use of representations or depictions of the feared situation or object (e.g., videos, photos, etc.) to aid imagination. For example, this might include a written script imagining touching a spider or speaking in front of the class or visiting the scene of the trauma.

Exposure can be conducted in a ‘graded’ manner where the feared situation or object is presented steps of gradually increasing difficulty. ‘Flooding’ entails exposing individuals with their most feared or anxiety-provoking scenario.

Exposure with response prevention involves individuals abstaining from compulsive or avoidance-related behaviours that are typically employed when confronted with fearful or anxiety-provoking situations, thoughts, or objects.

Exposure with eye movement typically involves directed eye movements (e.g., Eye Movement Desensitisation and Reprocessing [EMDR]) in facilitating the processing of fear-/anxiety- provoking memories.

Sometimes relaxation or breathing techniques are taught to be used in exposure conditions (e.g., systematic desensitisation). Similarly, individuals may also be encouraged to employ cognitive strategies (e.g., reframing, non-threat appraisals, etc.) during exposure practice.

Psychoeducation must entail explanation of the mechanisms underlying exposure therapy in reducing anxiety. It cannot be solely a description of the treatment itself (e.g., in experimental settings where participations are given a description of the task).

‘Technology-assisted’ exposure therapy involves using a computer-based or virtual reality program to aid with exposure practice. This kind of exposure can also use technology as a means to playback the exposure experience (e.g., audio or video recording).

‘Intensive’ exposure therapy was defined to be two or more hours of exposure practice within one session or having more than weekly exposure sessions.
